# Generalized Weighted Ordinal Pattern Descriptors for the Characterization of Adventitious Respiratory Sounds

**DOI:** 10.3390/e28101092

**Published:** 2026-10-07

**Authors:** Jorge I. Lopez Perez, Cristina D. Duarte, Claudio A. Delrieux

**Affiliations:** 1 Departamento de Ingeniería Eléctrica y Computadoras, Instituto de Ciencias e Ingeniería de la Computación, Universidad Nacional del Sur-Consejo Nacional de Investigaciones Científicas y Técnicas (CONICET), Bahía Blanca 8000, Argentina; cad@uns.edu.ar; 2 Departamento de Física, Instituto de Física del Sur, Universidad Nacional del Sur-Consejo Nacional de Investigaciones Científicas y Técnicas (CONICET), Bahía Blanca 8000, Argentina; cristina.duarte@uns.edu.ar

**Keywords:** ordinal patterns, permutation entropy, generalized weighted ordinal patterns, respiratory sound analysis, adventitious respiratory sounds

## Abstract

Automatic characterization of adventitious respiratory sounds is essential for developing computer-aided systems that support respiratory disease diagnosis. Commonly used acoustic descriptors, such as Mel frequency cepstral coefficients (MFCCs), primarily characterize spectral information and may not fully capture the nonlinear temporal dynamics of respiratory signals. In this work, we investigate the discriminative capability of Generalized Weighted Ordinal Pattern (GWOP) descriptors for representing adventitious respiratory sounds. Using respiratory recordings from the publicly available SPRSound pediatric database, GWOP descriptors were computed over multiple embedding dimensions, weighting factors, and time delays. An XGBoost classifier was employed exclusively as a supervised validation model to assess the discriminative information contained in the proposed feature representation, while the XGBoost Gain criterion was used to analyze the contribution of individual descriptor components. The proposed representation achieved stable performance across cross-validation folds, outperformed a conventional MFCC representation under the same evaluation protocol, and demonstrated that a reduced subset of Gain selected features preserved, and slightly improved, the classification performance while substantially reducing the dimensionality of the original feature space. These findings indicate that GWOP provides a lighter yet more informative and interpretable representation of adventitious respiratory sounds, capturing nonlinear ordinal dynamics that complement conventional spectral descriptors and offering a promising alternative for respiratory sound characterization.

## 1. Introduction

Respiratory diseases represent a major global health challenge, accounting for a substantial proportion of morbidity and mortality worldwide. According to worldwide studies, respiratory disorders remain among the leading causes of disability and premature death, generating a considerable socioeconomic impact on healthcare systems [1,2]. Therefore, early detection and accurate assessment of respiratory conditions are essential for timely diagnosis, disease monitoring, and treatment planning. Among the available diagnostic techniques, pulmonary auscultation remains one of the most accessible and widely used non-invasive methods because it provides valuable information about airflow and pathological alterations within the respiratory system. During auscultation, clinicians analyze normal and adventitious respiratory sounds, such as wheezes, crackles, rhonchi, and stridor, whose occurrence is associated with several respiratory disorders, including asthma, chronic obstructive pulmonary disease (COPD), pneumonia, and interstitial lung diseases [3,4,5].

Despite its clinical importance, conventional pulmonary auscultation is highly dependent on the clinician’s expertise and auditory perception, which may lead to subjectivity and inter-observer variability. Furthermore, respiratory sounds are inherently non-stationary signals whose characteristics vary according to multiple factors, including patient condition, anatomical recording location, respiratory phase, and acquisition device. The coexistence of multiple adventitious events within the same respiratory cycle, together with environmental and physiological noise, further increases the difficulty of their interpretation. These limitations have motivated the development of computer-aided respiratory sound analysis systems capable of providing objective, reproducible, and quantitative assessments to support clinical decision making [4,6].

Automatic respiratory sound analysis has received increasing attention during the last decade. Existing computational approaches can be broadly categorized into handcrafted acoustic descriptors, time frequency representations, and deep learning based methods [6,7]. Traditional features, including Mel-frequency cepstral coefficients (MFCCs), spectral descriptors, and wavelet-based representations, have been extensively investigated because of their computational efficiency and relatively straightforward interpretation [8,9,10]. In parallel, the availability of annotated respiratory sound databases, such as the International Conference on Biomedical and Health Informatics (ICBHI) respiratory sound database and the SPRSound dataset, has enabled the development of increasingly sophisticated machine learning and deep learning approaches [11,12]. Convolutional neural networks and other deep architectures have demonstrated competitive performance by automatically learning discriminative representations from respiratory recordings [13,14]. However, these approaches generally require large amounts of labeled data, considerable computational resources, and often provide limited interpretability regarding the relationship between learned features and physiological mechanisms.

Respiratory sounds are generated through complex interactions between airflow turbulence, airway geometry, tissue mechanics, and pathological alterations in the respiratory system [3,15]. As a result, they exhibit rich temporal dynamics that cannot be fully characterized by conventional linear or spectral descriptors alone, which motivates researching the applicability of nonlinear time-series analysis in this context. Early evidence came from fractal analysis, where Oliveira et al. [16] showed that normal lung sounds exhibited a nearly constant time-dependent fractal dimension (≈1.49), whereas recordings containing adventitious sounds displayed pronounced temporal variations and multiple local fractal dimensions. This finding suggested that pathological conditions alter the underlying dynamics rather than only their spectral content. Building on these findings, Gnitecki and Moussavi [17] demonstrated that inspiratory lung sounds exhibit self-affine behavior, a hallmark of fractal time series, meaning that their statistical structure is preserved across temporal scales. A stronger justification for nonlinear analysis was later provided in [18], where the authors explicitly investigated whether respiratory sounds satisfy the assumptions required for nonlinear dynamical analysis. Using recurrence plots, surrogate-data testing, and chaotic invariants, they provided evidence consistent with nonlinear structure. This evidence has established nonlinear time-series analysis as a suitable framework for respiratory sound characterization, enabling the quantification of complexity and temporal organization beyond conventional frequency-domain analysis.

Among complexity measures, Permutation Entropy (PE), introduced by Bandt and Pompe [19], has become one of the most widely used approaches for analyzing biomedical signals due to its simplicity, computational efficiency, robustness to noise, and invariance to monotonic transformations [20,21]. Unlike conventional spectral measures, PE does not rely on signal amplitude values. Instead, it characterizes the probability distribution of ordinal patterns generated from the temporal ordering of neighboring samples. This property has enabled its successful application in several biomedical domains, including electroencephalography, electrocardiography, heart rate variability, and eye-tracking signals, where complexity assessment provides relevant information indistinguishable by other analysis techniques [21,22,23,24]. Entropy-based descriptors have also shown promise in the specific context of respiratory sound analysis. For instance, multiscale permutation entropy has been reported to improve lung sound classification accuracy over other entropy variants [25], and Shannon, logarithmic, and spectral entropy features have been combined with ensemble classifiers for multi-class identification of respiratory diseases from stethoscopic recordings [26]. Since ordinal patterns are determined exclusively by the relative values of consecutive samples, the encoding process is oblivious to the actual amplitudes.

## 2. Materials and Methods

**Figure 1:**
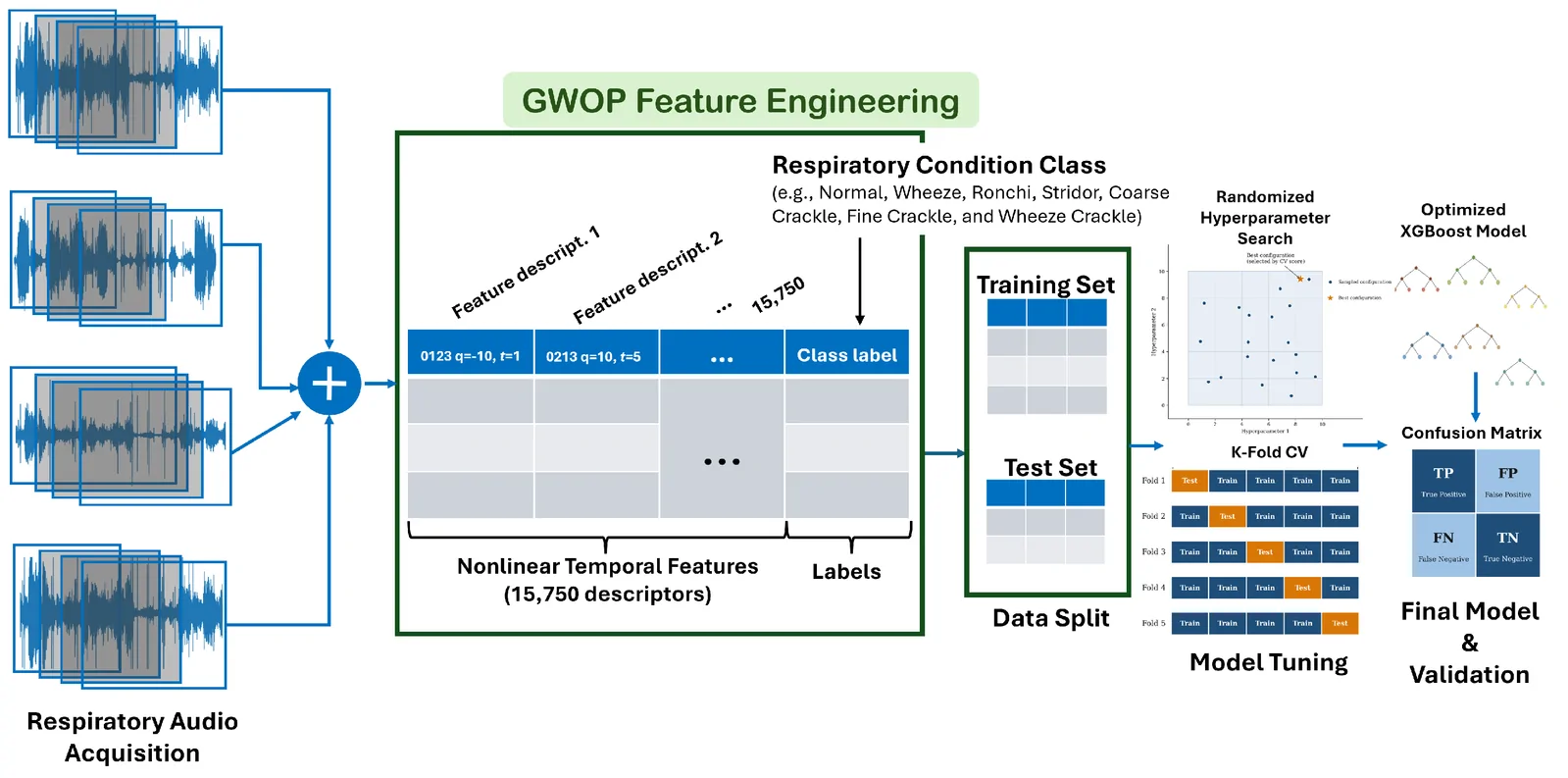
Overview of the proposed framework for respiratory sound classification using Generalized Weighted Ordinal Pattern (GWOP) feature extraction and XGBoost-based modeling.

Figure 1 summarizes the proposed methodological framework for respiratory sound analysis. The workflow begins with the acquisition of respiratory audio recordings, which are segmented using a windowing strategy to generate individual analysis frames. Subsequently, the Generalized Weighted Ordinal Pattern (GWOP) method is applied to extract nonlinear temporal features from each respiratory sound segment, resulting in a high-dimensional feature representation composed of 15,750 descriptors. The extracted feature vectors are then divided into training and testing subsets to evaluate the classification performance. Hyperparameter optimization is performed using a randomized search strategy combined with 5-fold cross-validation to identify the optimal configuration of the classifier. Finally, the optimized XGBoost model is trained and evaluated using the independent test set, and its performance is assessed through standard classification metrics.

### 2.1. Respiratory Sound Database

**Figure 2:**
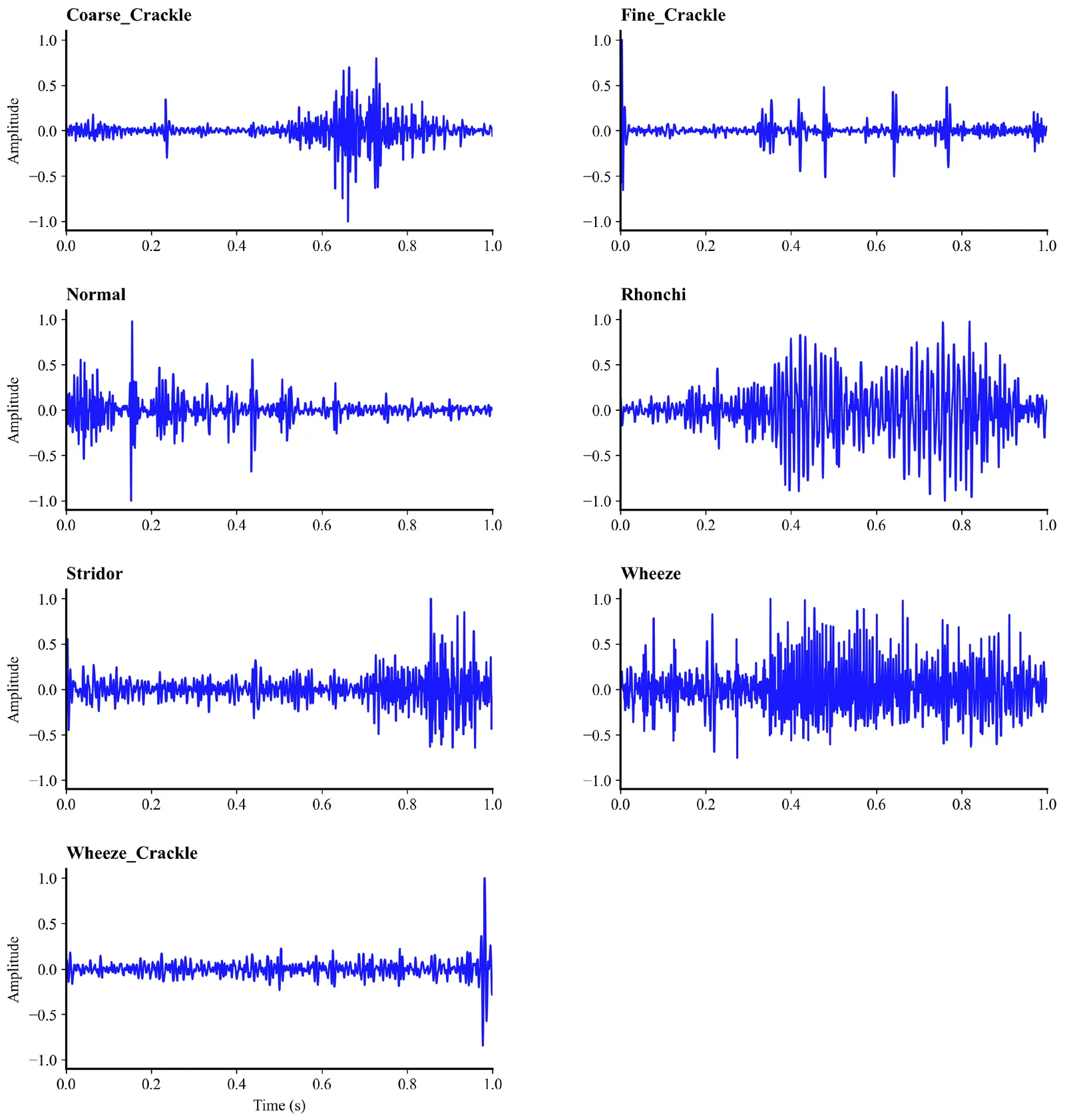
Representative waveforms of respiratory sound recordings from each type of event in the SPRSound database.

This study made use of the SPRSound database (Open-Source SJTU Pediatric Respiratory Sound Database) [12], the first publicly available open-access respiratory sound database recorded exclusively in the pediatric population, comprising patients aged between 1 month and 18 years. Recordings were obtained at the Pediatric Respiratory Department of Shanghai Children’s Medical Center (SCMC, Shanghai, China) using Yunting Model II stethoscopes and stored in .wav format with a sampling frequency of 8 kHz. The database provides annotations at two complementary levels. At the record level, each recording is labeled as Normal (N), Continuous Adventitious Sounds (CAS), Discontinuous Adventitious Sounds (DAS), CAS & DAS, or Poor Quality. At the event level, individual respiratory events are annotated with their temporal boundaries (onset and offset in milliseconds) and classified as Normal (N), Rhonchi (R), Wheeze (W), Stridor (S), Coarse Crackle (CC), Fine Crackle (FC), or Wheeze & Crackle (WC). For the present analysis, the training partition of the 2022 to 2025 release was used, comprising 6641 recordings. Recordings flagged as Poor Quality at the record level were excluded from the analysis. From the remaining files, a sliding-window approach was applied to extract overlapping segments of 1 s at the native sampling rate of each recording. Segments were assigned to one of seven classes according to the event-level annotation whose temporal span fully covered the segment. Representative waveforms of each class are shown in Figure 2.

### 2.2. Nonlinear Feature Extraction

**Figure 3:**
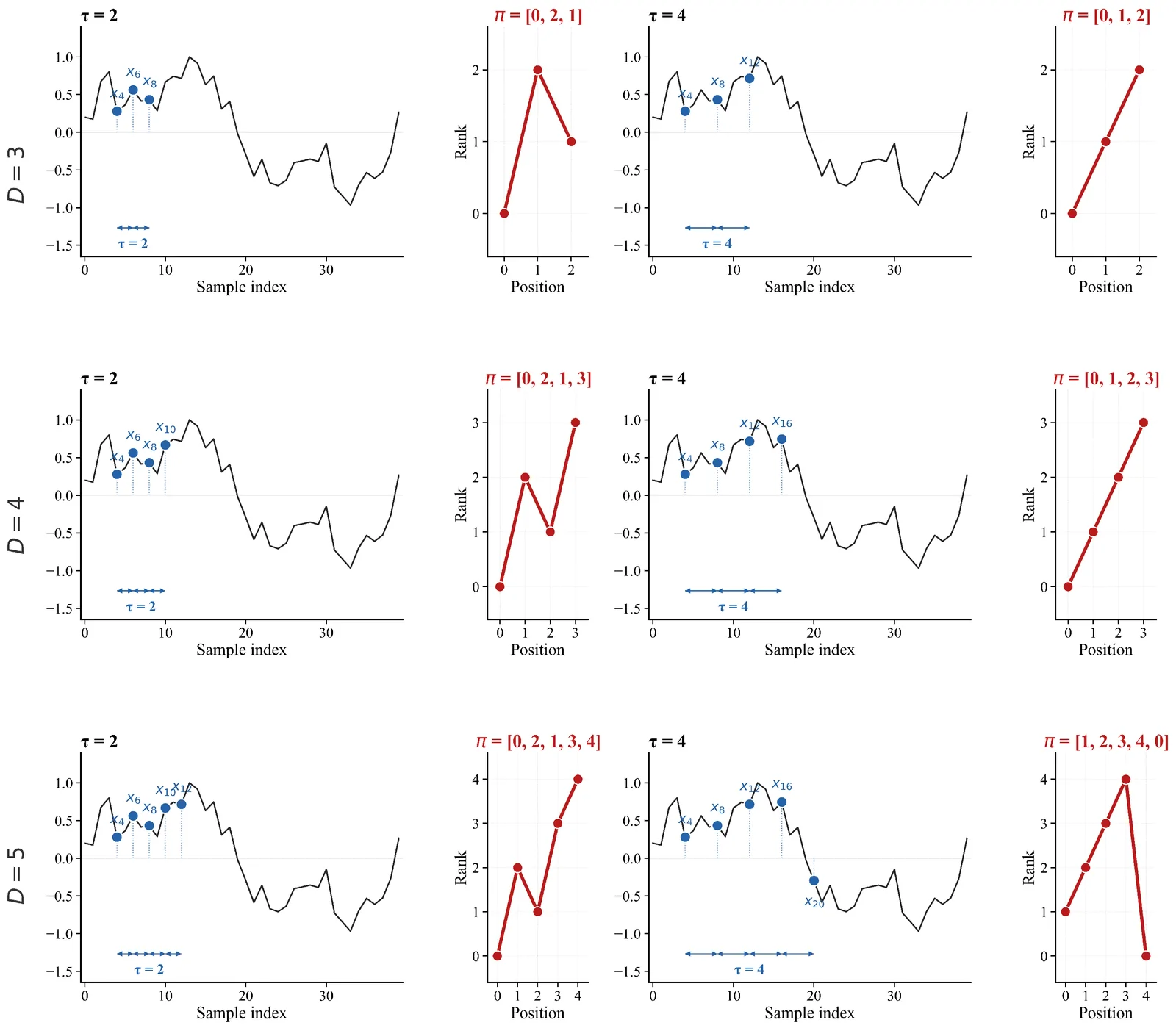
Effect of time delay τ on ordinal pattern construction for different embedding dimensions.

To quantify the underlying dynamics of respiratory sounds, we employed an extension of the symbolic representation of time series through ordinal patterns (OPs), introduced by Bandt & Pompe [19]. In this representation, time series are encoded into symbols that capture the rank-order relationships among consecutive signal samples, providing a robust and compact description [27]. The diversity and uniformity of the relative frequencies of these OPs along a time series can be effectively used to characterize its regularity (or lack thereof). Beyond this, the ordinal probability distribution also carries information that helps separate deterministic chaotic dynamics from stochastic fluctuations, for instance through the entropy–complexity plane [27] or through more recent ordinal-based quantifiers [28]. Given a time series $X=\{x_{1},x_{2},\ldots ,x_{T}\}$ of length *T*, the OPs are constructed by extracting overlapping segments $v_{s}$:

(1) $$v_{s}=\left(x_{s},\;x_{s+\tau },\;x_{s+2\tau },\;\ldots ,\;x_{s+(D-1)\tau }\right),$$

where the embedding dimension *D* determines how many samples are included in each segment, and the time delay τ specifies the temporal spacing between them. A total of N=T−(D−1)τ segments are extracted from the original series *X*. Within each segment, the *D* values are assigned ordinal labels (0,1,…,D−1) according to their rank-order relationships. Conflicts arising in case of identical sample values (unlikely but possible in biological signals) can be resolved either by deterministic or random schemes, with no actual effect on the analysis results. In total, there exist D! possible permutations π to which a segment of the signal can be mapped. Figure 3 presents examples of OPs for D=3,4,5, illustrating the effect of varying the delay parameter τ as well. The relative frequency (empirical probability) of each $\pi_{i}$ is defined as:

(2) $$p\left(\pi_{i}\right)=\frac{\sum_{s:\;\pi_{s}=\pi_{i}}1}{\sum_{s}1},$$

where $\pi_{s}$ denotes the OP corresponding to segment *s*. For a robust empirical probability evaluation, it is recommended that N>5D!. The probability distribution over all D! possible patterns is then defined as $P_{\pi }=\left\{p\left(\pi_{1}\right),\ldots ,p\left(\pi_{D!}\right)\right\}$.

However, using only rank-order information makes the method oblivious to relative amplitudes, which can be disadvantageous in several contexts. For this reason, Weighted Permutation Entropy assigns higher contribution to patterns with larger amplitude ranges, thus preserving information related to signal variability while maintaining the advantages of ordinal analysis [29]. More recently, Generalized Weighted Permutation Entropy (GWPE) extended this concept by introducing an entropic index that improves the characterization of complex nonlinear signals [30]. This entropic index *q* plays a role analogous to the *q*-parameter of non-extensive entropies (Tsallis and Renyi), extending the classical entropy analysis toward a generalized framework that can distinguish between different fluctuation regimes. The generalized weighted ordinal pattern (GWOP) probability distribution $P_{\pi }^{\left(q\right)}=\left\{p^{\left(q\right)}\left(\pi_{1}\right),\ldots ,p^{\left(q\right)}\left(\pi_{D!}\right)\right\}$ is thus defined as

(3) $$p^{\left(q\right)}\left(\pi_{i}\right)=\frac{\sum_{\left\{s\;:\;\pi_{s}=\pi_{i}\right\}}w_{s}^{\left(q\right)}}{\sum_{s}w_{s}^{\left(q\right)}},$$

where

(4) $$w_{s}^{\left(q\right)}={\left(\frac{1}{D}\sum_{k=0}^{D-1}{\left[x_{s+k\tau }-{\bar{v}}_{s}\right]}^{2}\right)}^{\;q/2},$$

and ${\bar{v}}_{s}=\frac{1}{D}\sum_{k=0}^{D-1}x_{s+k\tau }$ is the mean of segment $v_{s}$. Segments with zero range (locally constant signal) yield $w_{s}^{\left(q\right)}\to \infty$ for q<0. In such cases the weight is set to zero to avoid numerical indeterminacy [30]. The parameter q∈R controls the sensitivity of the weighting to the scale of the fluctuations: for q>0, the importance of higher range OPs is amplified, and conversely for q<0 low range OPs are emphasized. It is worth noting that for q=0, GWOP collapses into the Bandt & Pompe OP distribution. No envelope normalization or any other pre-processing of the raw signal was applied prior to GWOP estimation. This is a deliberate design choice: the study aims to assess the raw sensitivity of ordinal patterns to adventitious sound structure without imposing an *a priori* assumption about which amplitude components are informative.

### 2.3. Feature Extraction

Since GWOP descriptors are computed from short temporal windows, the original recordings from the SPRSound dataset (2022–2025) were partitioned into fixed-length segments using a sliding-window approach. The use of one-second windows limits the temporal variability within each segment, providing a more reliable estimation of the corresponding ordinal pattern distributions while preserving the local dynamics of the respiratory signal. Each audio file was divided into one-second segments with a 50% overlap (500 ms hop size), yielding two windows per second of recording. Windows were labeled according to the event annotations provided in the dataset’s JSON files, with each window assigned the label of the annotation exhibiting the greatest temporal overlap. Windows with no overlapping annotated event were labeled as Normal. Audio files shorter than one second were zero-padded to ensure a uniform segment length prior to GWOP feature extraction.

This procedure produced 17,682 one-second segments distributed across seven classes. To mitigate class imbalance without introducing synthetic samples, an adaptive sampling strategy was employed by randomly downsampling the majority classes (Normal, Wheeze, and Fine_Crackle), while the minority classes (Coarse_Crackle, Rhonchi, Stridor, and Wheeze_Crackle) were retained at their original size, resulting in a moderately imbalanced dataset of 8991 segments. A fixed random seed (42) was used for the downsampling procedure for reproducibility. Table 1 summarizes, for each class, the number of original samples, the balancing strategy applied, and the final class distribution. No additional digital filtering was applied before feature extraction to preserve the original GWOP structure of the respiratory signals.

Each one-second respiratory segment obtained during the segmentation stage was independently processed using the GWOP descriptor. For each segment, GWOP distributions were computed across multiple embedding dimensions, entropic indices, and temporal delays. Specifically, embedding dimensions D∈{3,4,5} were considered. The weighting parameter was evaluated in the range q∈[−10,10], resulting in 21 different weighting configurations, while the time delay parameter was evaluated as τ∈{1,2,3,4,5}, resulting in five temporal configurations. The descriptors obtained from all combinations of these parameters were concatenated to form a single fixed-length feature representation. The dimensionality of the GWOP feature vector is thus $\sum_{D\in \{3,4,5\}}D!\times 21\times 5=\left(6+24+120\right)\times 21\times 5=15750$. Accordingly, each respiratory segment was represented by a fixed-length 15,750-dimensional GWOP feature vector. This high dimensional representation preserves the nonlinear ordinal dynamics captured across multiple embedding dimensions, weighting factors, and temporal delays, providing a comprehensive characterization of each respiratory segment. Consequently, the final balanced dataset contains 8991 respiratory sound segments and 15,750 GWOP features.

### 2.4. Classification

An eXtreme Gradient Boosting (XGBoost) model was trained to automate the respiratory sound classification. XGBoost [31] is an ensemble learning method that constructs an additive sequence of decision trees by minimizing a regularized objective function through a weighted second-order gradient optimization. This particular classification model was selected given that its intrinsic feature importance measure (based on gradient gain) enables post-hoc analysis of the contribution of individual GWOP features and their aggregation according to the (D,q,τ) parameters. This provides interpretable insights into the discriminative characteristics captured by the ordinal representation. Also, the computational efficiency of the model makes it suitable for the high-dimensional feature spaces [32]. At the same time, support for sample weighting allows class imbalance to be explicitly addressed during training without requiring additional resampling procedures. As mentioned above, majority classes were reduced through random downsampling (see Table 1), and the residual imbalance, ranging from 306 instances for Stridor to 2038 for Normal, was further addressed during training through inverse-frequency sample weighting. Thus, to mitigate potential biases, each training sample was weighted according to the inverse frequency of its corresponding class, allowing the classifier to assign greater importance to minority classes during the learning process [33,34].

The 8991 labeled windows were partitioned into a training set (80 %, n=7192) and a held-out test set (20 %, n=1799) using a stratified random split, preserving the original class proportions in both subsets. The held-out (test) set was used for final performance evaluation and was never exposed during model development. The XGBoost hyperparameters were set as follows: number of estimators n_estimators =500; maximum tree depth max_depth =8; learning rate learning_rate =0.03; row subsampling subsample =0.7; column subsampling per tree colsample_bytree =0.7; multiclass objective objective = multi:softprob; evaluation metric eval_metric = mlogloss. These values were selected based on established practice for high-dimensional tabular data [31] and kept fixed to provide a reproducible baseline that isolates the contribution of the GWOP feature representation. All experiments were conducted using Python 3.x and the XGBoost library. Model training and evaluation were performed on a workstation equipped with an NVIDIA GeForce RTX 5070 Ti GPU with 12 GB of VRAM, 32 GB of system memory, and an Intel Core Ultra 7 255HX processor. This hardware configuration provided sufficient computational resources for training the gradient-boosted decision tree models on the high dimensional GWOP feature matrix.

## 3. Results

This section presents the experimental validation of the proposed GWOP descriptor for respiratory sound analysis. The evaluation is designed to assess the discriminative capability of the entropy-based representations generated from respiratory sound segments. The analysis is organized into four main stages: (i) baseline evaluation of the GWOP feature representation using an XGBoost classifier as a supervised validation tool, (ii) assessment of the effect of hyperparameter optimization on the reliability of the descriptor evaluation, (iii) ablation analysis of the Gain-based feature selection strategy to identify the most informative GWOP components and facilitate the interpretation of the extracted representations, and (iv) comparison of the GWOP representation with conventional spectral features for respiratory sound classification.

### 3.1. Baseline Validation of GWOP Representations

The discriminative capability of the proposed GWOP feature representation was initially assessed using an XGBoost classifier as a supervised evaluation tool. A 5-fold cross-validation strategy was applied on the training feature set to quantify the consistency of the information encoded by the entropy based descriptors. As shown in Table 2, the obtained results exhibit stable performance across all folds, achieving a mean accuracy of 0.4695 (±0.0120) and a macro F1-score of 0.4571 (±0.0190). The low variability among folds indicates that the extracted GWOP representations provide consistent class related information and that the observed performance is not dependent on a specific data partition. These results establish a reliable baseline for analyzing the contribution of the different GWOP configurations and feature selection strategies investigated in the following experiments.

### 3.2. Effects of Hyperparameter Optimization

**Figure 4:**
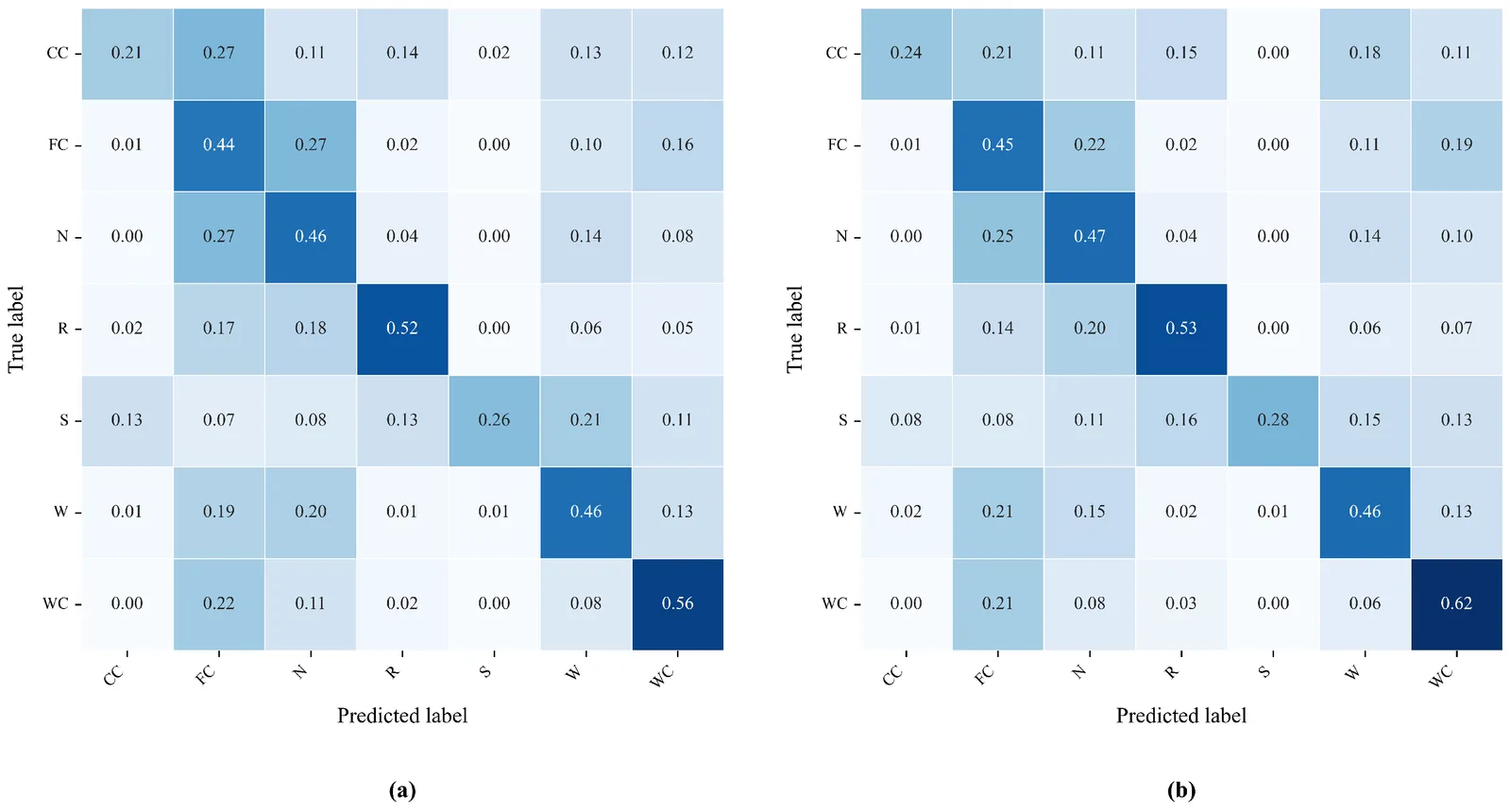
Normalized confusion matrices for (**a**) the baseline, and (**b**) the optimized XGBoost classifier. Each cell shows the proportion of samples of a true class (rows) assigned to a predicted class (columns); darker shades indicate higher proportions. The corresponding confusion matrices in absolute sample counts are provided in Figure S1 of the Supplementary Materials.

Figure 4 compares the normalized confusion matrices obtained using the baseline and optimized XGBoost configurations as validation models for the GWOP representation. The optimization process maintains the general discrimination pattern provided by the entropy-based descriptors while producing moderate improvements in class-wise recognition. In particular, the optimized configuration leads to a slight increase in recall for all classes except Stridor. The largest improvement is observed for Wheeze_Crackle, with recall increasing from 56% to 62%. The confusion patterns remain consistent between both configurations, highlighting the intrinsic difficulty of separating specific respiratory sound categories. Fine_Crackle and Normal samples exhibit mutual confusion, suggesting overlapping acoustic characteristics that are not completely resolved by the GWOP representation. Likewise, Wheeze samples show considerable similarity with Fine_Crackle and Normal classes.

### 3.3. Analysis of Informative GWOP Components via Gain-Based Feature Selection

**Figure 5:**
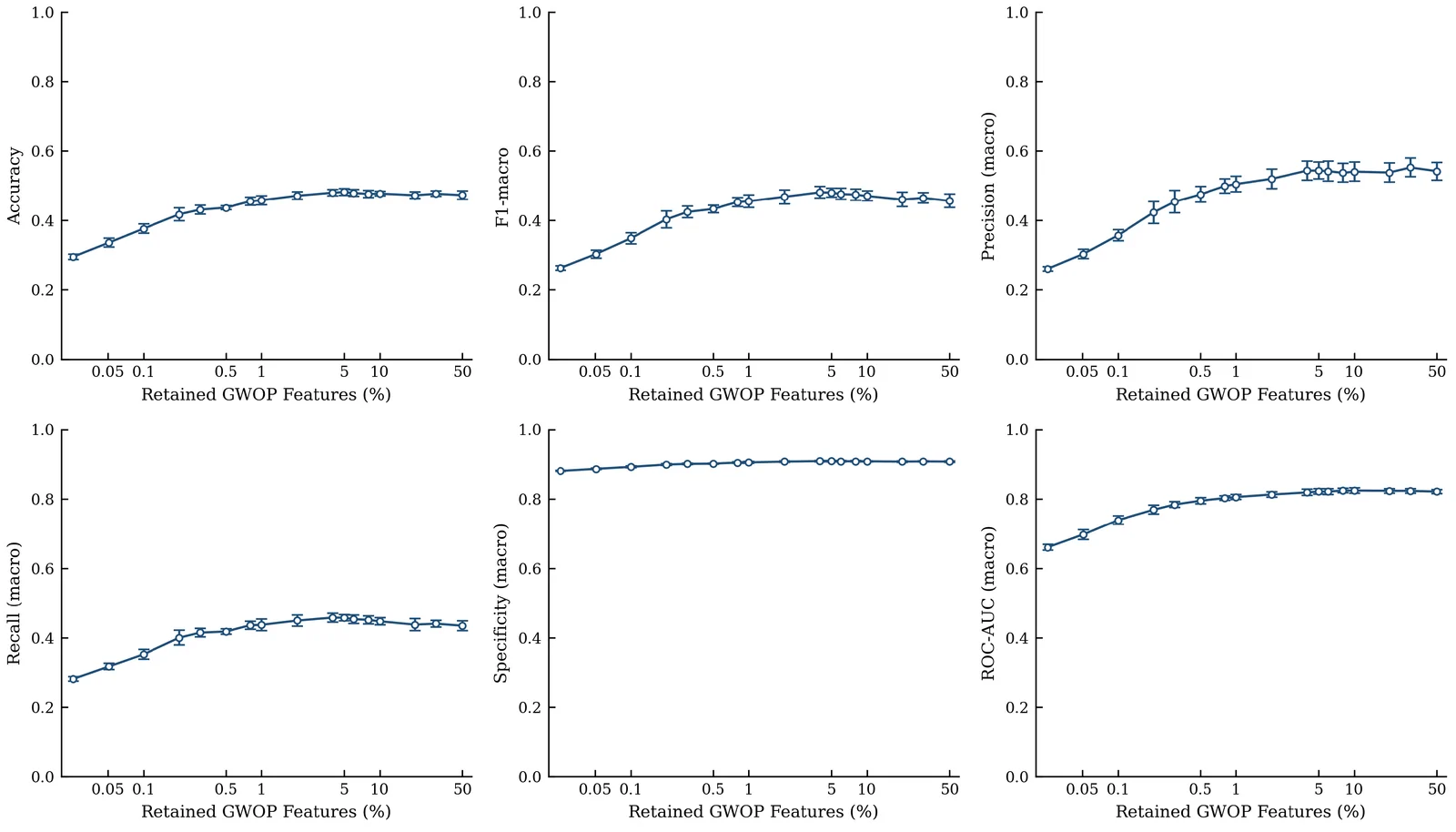
Cross-validation performance across GWOP feature subsets of varying size, retained according to their individual XGBoost Gain ranking. Each panel reports a different macro-averaged classification metric as a function of the percentage of GWOP features retained (log scale). Points indicate the mean value across the five folds of the cross-validation procedure, and error bars indicate the corresponding standard deviation.

An additional analysis was conducted to investigate the contribution of individual GWOP components and identify the most informative features within the high-dimensional entropy-based representation. A Gain-based feature selection strategy was applied to quantify the relative importance of each descriptor and evaluate whether the dimensionality of the GWOP representation could be reduced while preserving its discriminative capability. We ranked the features based on cumulative gain and then progressively reduced the feature set by retaining decreasing percentages of the total number of features. The feature set size was progressively reduced from 50% to 0.025% in logarithmic steps. Table 3 and Figure 5 present the evaluation results obtained using the top-ranked retained features based on the cumulative gain.

The complete GWOP representation, comprising 15750 features, achieved a macro-F1 score of 0.4758, with a macro-specificity of 0.9086, macro-precision of 0.5665, and macro-sensitivity of 0.4507. Progressive reductions from 100% to 1% of the top features did not result in a noticeable degradation in the classification performance. In fact, the best performance was obtained when retaining the top 10% of the features, with a macro-F1 score of 0.4977, macro-specificity of 0.9112, macro-precision of 0.5695, and macro-sensitivity of 0.4732. For thresholds below 0.1% of the top features, all evaluation metrics decreased by approximately 0.1 points compared with the values observed at higher thresholds.

**Figure 6:**
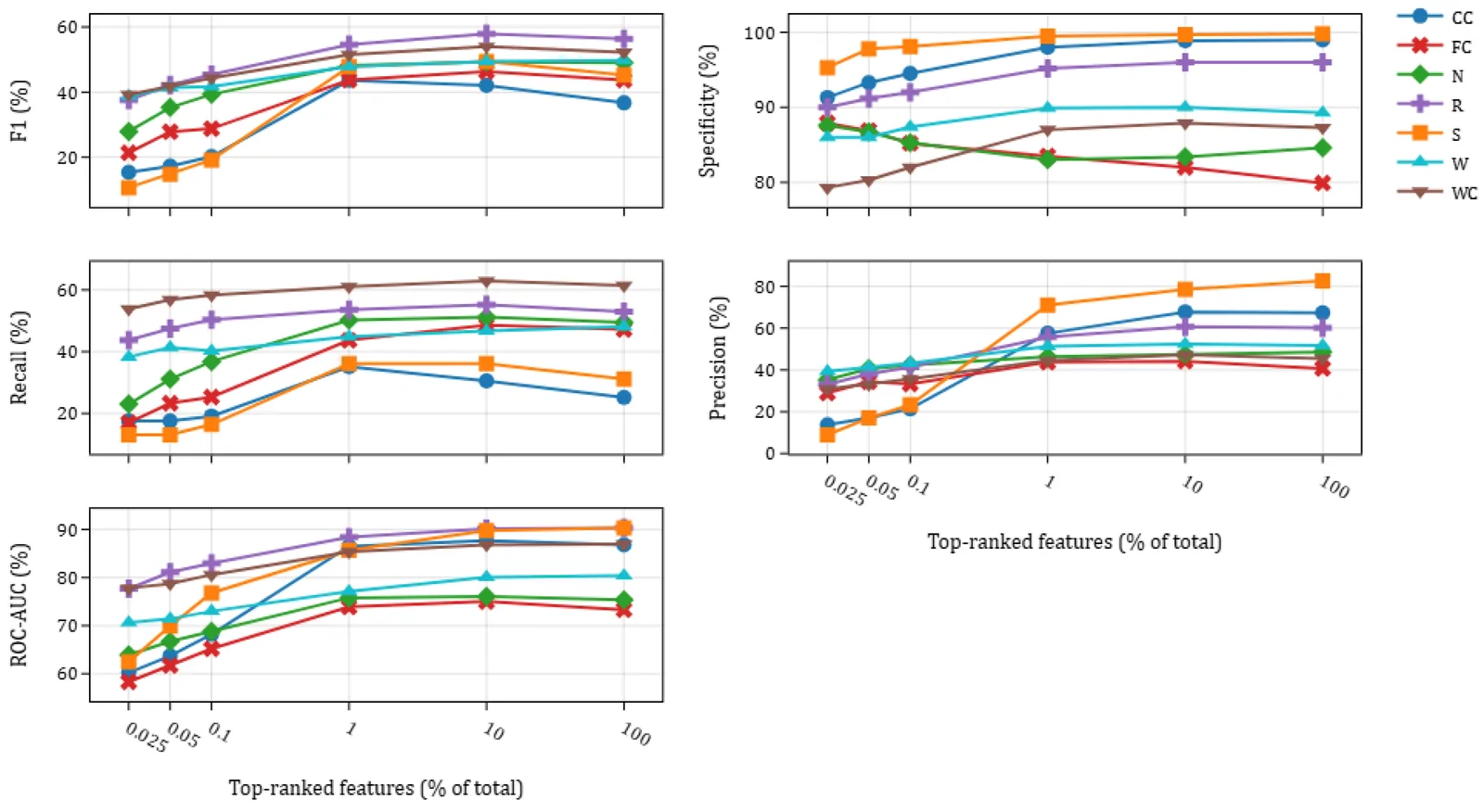
F1, specificity, recall, and precision metrics for respiratory sound classification separated by classes across reduced sets of top-ranked features.

**Figure 7:**
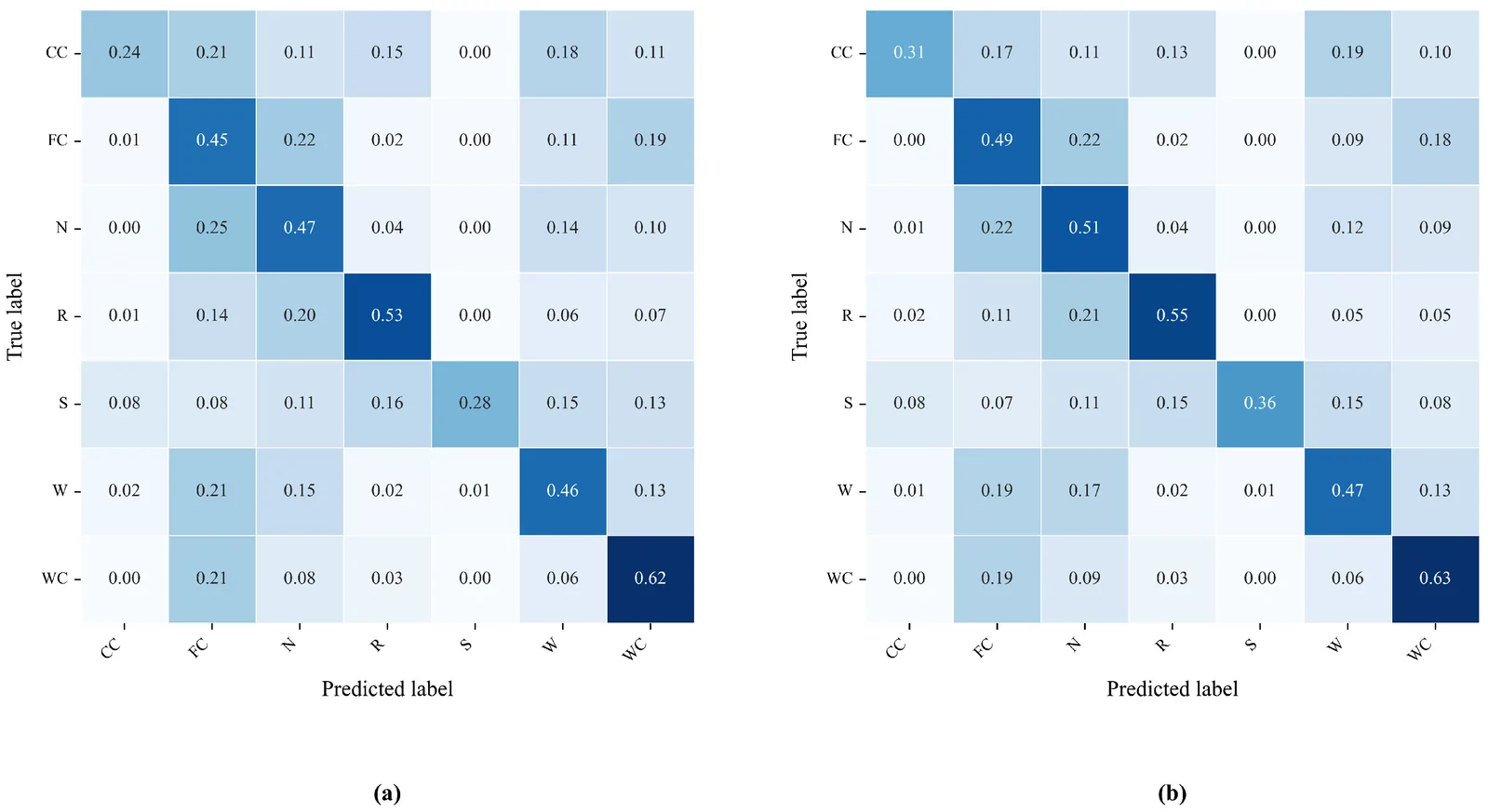
Normalized confusion matrices obtained using the complete and Gain-selected GWOP representations. The left panel (**a**) corresponds to the complete GWOP feature representation (100%, 15,750 features), while the right panel (**b**) corresponds to the top-10% Gain-selected subset (1575 features), ranked by individual feature Gain. Darker shades indicate higher proportions. The corresponding confusion matrices in absolute sample counts are provided in Figure S2 of the Supplementary Materials.

The same ablation approach was used to assess how the performance of individual classes changed as the number of retained features was reduced. The per-class performance metrics are shown in Figure 6. To further investigate the internal organization of the most informative GWOP descriptors, the 10% highest-ranked features according to the gain importance of the complete model were analyzed in terms of the (D,q,τ) parameters (see Table 4). Figure 7 complements this analysis by comparing the normalized confusion matrices obtained using the complete GWOP representation and the top-10% Gain-selected subset.

### 3.4. Comparison with Conventional Spectral Features

To further assess the discriminative capability of the proposed GWOP representation, its performance was compared with a conventional spectral representation based on Mel-Frequency Cepstral Coefficients (MFCCs), which are widely used for respiratory sound analysis. For each 1-s segment sampled at 8 kHz, 20 MFCC coefficients were extracted using a 512-point FFT, a hop length of 256 samples, and a Hann window. The coefficients obtained from all analysis frames were concatenated into a single feature vector. Both MFCC and GWOP representations were evaluated using the same train/test partition and the same optimized XGBoost configuration to ensure a fair comparison between feature spaces.

Table 5 summarizes the evaluation results obtained with both representations. The GWOP descriptor achieved superior overall discrimination compared with MFCCs. Cohen’s kappa increases from 0.2874 to 0.3923, a relative improvement of 36% compared to the 20% relative gain in accuracy; since kappa discounts agreement expected by chance, this indicates that the improvement is not attributable to the class distribution. Balanced accuracy also increases more than accuracy (0.3750 to 0.4796 against 0.4202 to 0.5053), confirming that the gain is concentrated in the minority classes. Finally, the macro AUC rises from 0.7860 to 0.8383, indicating greater class separability in the feature space rather than a more favorable decision threshold. This consistent advantage indicates that the ordinal information captured by GWOP provides additional class-related information beyond that contained in conventional spectral features.

**Figure 8:**
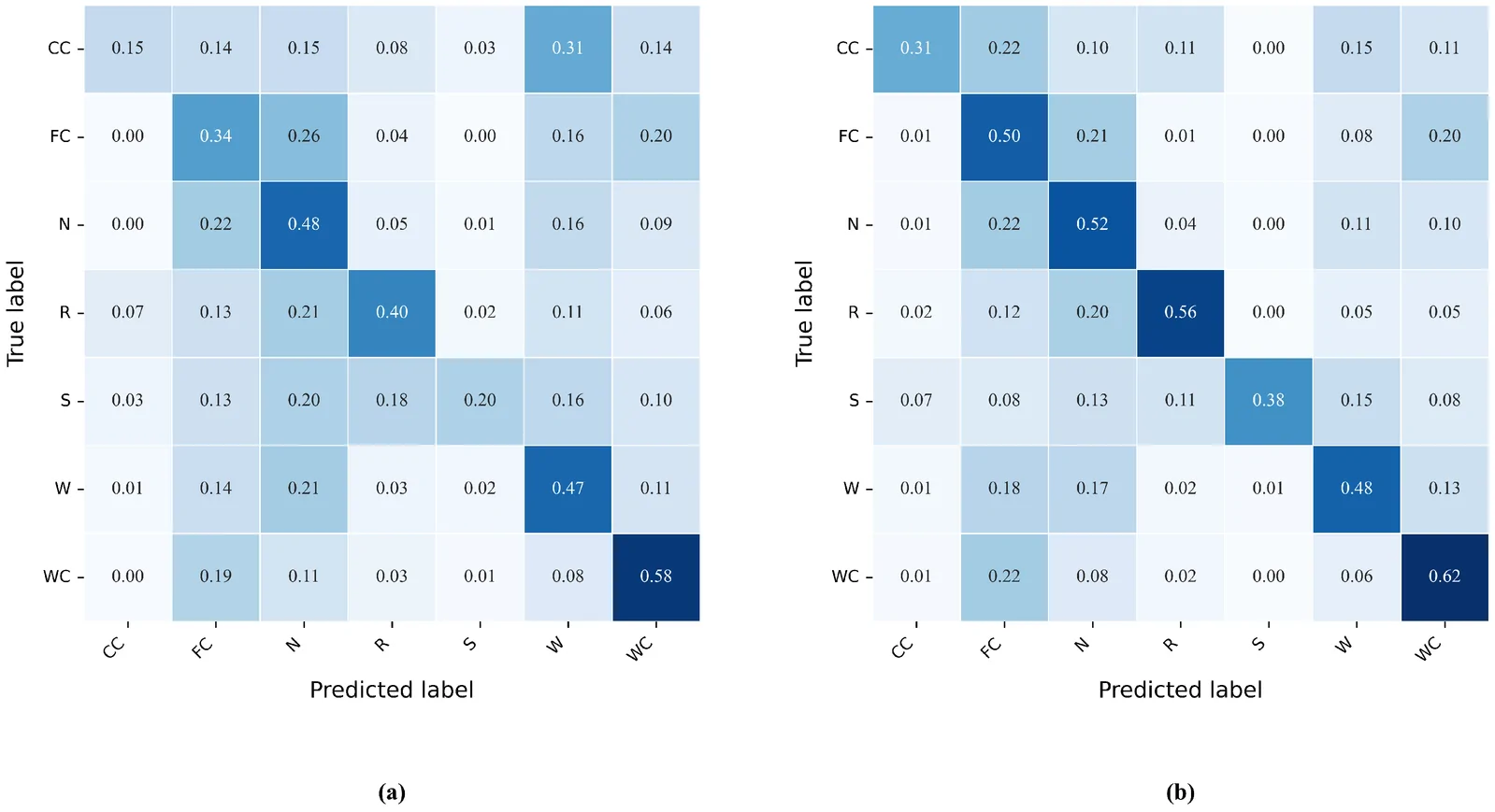
Normalized confusion matrices obtained using (**a**) the conventional MFCC representation and (**b**) the proposed GWOP descriptor with Gain-based Top-10% feature selection, evaluated with the optimized XGBoost model as a common validation classifier. GWOP provides a more favorable classification pattern across all adventitious respiratory sound classes. Darker shades indicate higher proportions. The corresponding confusion matrices in absolute sample counts are provided in Figure S3 of the Supplementary Materials.

The class-wise analysis shows that GWOP improves the F1-score for all respiratory sound categories, with gains ranging from 0.02 for Wheeze_Crackle to 0.26 for Stridor. The largest improvement is observed for Coarse Crackle and Stridor, where the F1-score increases from 0.23 to 0.42 and 0.26 to 0.51 respectively, due to simultaneous gains in precision and recall. Similar improvements are obtained for Fine Crackle, Rhonchi, and Wheeze, suggesting that the entropy-based ordinal representation is particularly effective for capturing transient acoustic structures associated with adventitious respiratory events. Figure 8 compares the normalized confusion matrices obtained with both feature representations. The overall confusion structure remains similar, indicating that some class ambiguities are associated with intrinsic acoustic similarities between respiratory sound categories rather than exclusively with the selected representation. Nevertheless, the GWOP-based representation exhibits improved diagonal concentration for all adventitious sound classes, supporting the quantitative improvements observed in Table 5. In the particular case of Coarse Crackle, the conventional representation shows a substantial confusion with Wheeze, with 31% of Coarse Crackle samples classified as Wheeze. In contrast, the GWOP representation reduces this confusion to 15%, although this is accompanied by an increase in the misclassification of Coarse Crackle as Fine Crackle, from 14% to 22%, so that the residual error shifts from Wheeze to Fine Crackle. Overall, the recall for Coarse Crackle increases from 15% to 31%. For Stridor, the conventional representation shows a strong confusion with the Normal class, with only 20% of Stridor samples correctly classified and a similar proportion misclassified as Normal. This is compounded by a precision of 0.36, indicating that the classifier assigns this label with little discrimination. With GWOP, both aspects improve: recall rises to 38% and precision to 0.79, the largest gain of any class in Table 5. The model therefore becomes highly reliable when it predicts Stridor, despite this being the least represented class in the dataset (*n* = 306).

### 3.5. Ablation Tests with Subject-Wise Splitting

**Figure 9:**
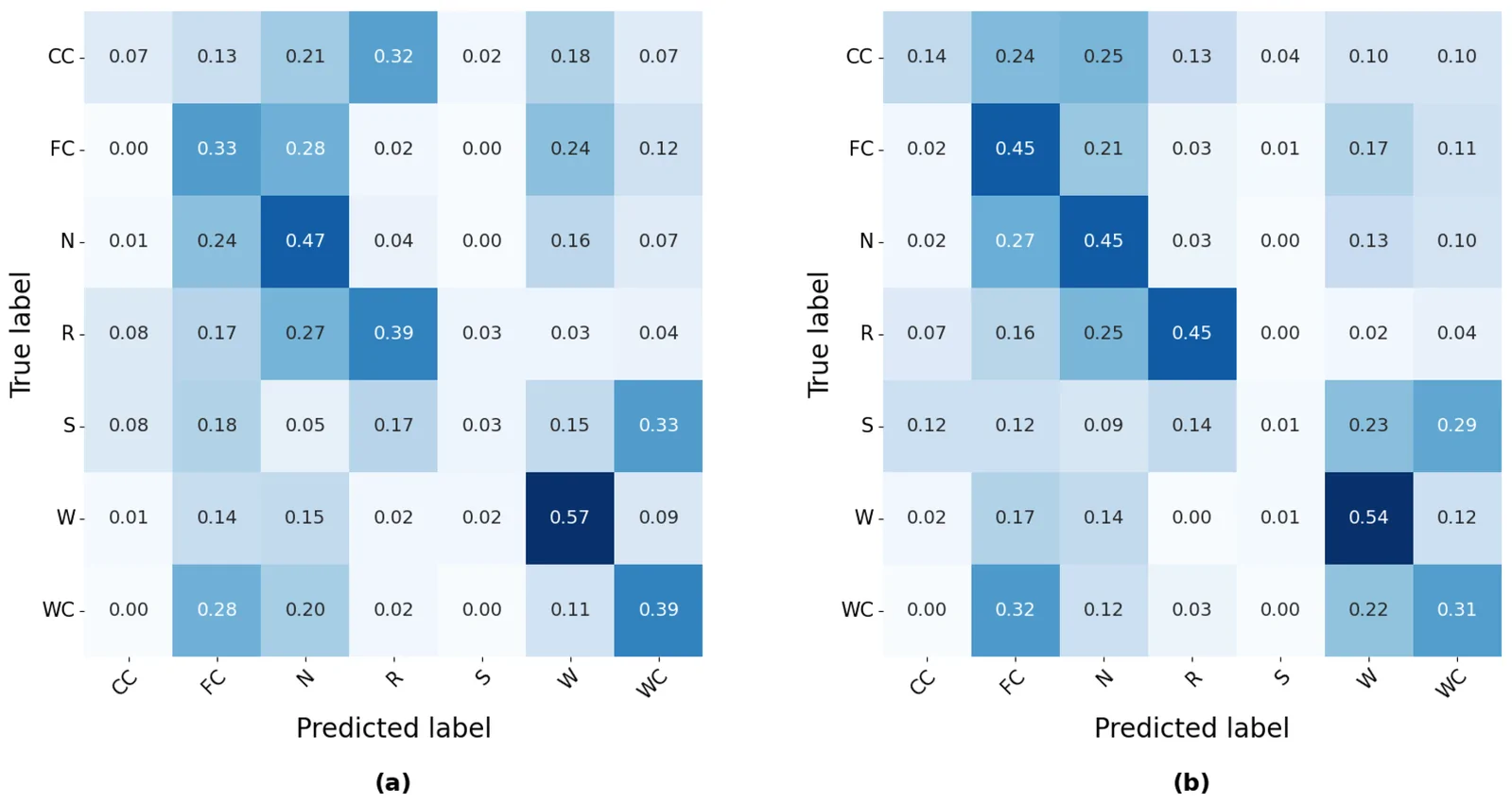
Normalized confusion matrices for the subject-wise classification models: (**a**) MFCC and (**b**) GWOP. Darker shades indicate higher proportions.

As is the case in most biosignals, there exists a tendency to identify individuals from distinctive, subject-specific patterns embedded in physiological recordings, even when these signals are not explicitly intended for identification. Even though this fingerprinting phenomenon is not explicitly reported in the literature of adventitious respiratory sound (as in other biosignals such as EEG, ECG, EMG, and fMRI), we conducted an ablation test in which a subject-wise splitting was performed to avoid the possibility of data leakage during model training. For this, the 783 participating subjects were split into a training group of 627 subjects and a test group of 156 subjects (approximately 80–20%), with a uniform proportion of participants per age strata. Table 6 reports the number of subjects and segments per age group, and Table 7 the number of segments per class in each subset. The 8991 audio segments of each group were used respectively to model training and model testing. Because the split was stratified by subject age rather than by segment class, the fraction of segments assigned to the test set varies across classes and across age groups. The normalized confusion matrices of this experiment (both for GWOP-based and MFCC classifiers) are presented in Figure 9.

## 4. Discussion

The experimental results demonstrate that the proposed GWOP-based entropy representation provides a meaningful and discriminative feature space for respiratory sound characterization. The stable performance across cross-validation folds (accuracy: 0.4695±0.0120; macro-F1: 0.4571±0.0190) indicates that the extracted descriptors capture reproducible acoustic patterns rather than characteristics dependent on a particular data partition. Although hyperparameter optimization resulted in moderate improvements, the overall confusion structure remained largely unchanged, suggesting that the remaining ambiguities are primarily associated with intrinsic similarities between respiratory sound categories rather than with the specific configuration of the validation classifier. These persistent confusion trends indicate that the observed limitations may be related to the complexity of the underlying acoustic phenomena rather than solely to the classifier configuration.

The gain-based feature selection provides compelling evidence for the informational efficiency of the GWOP representation. Reducing the feature space from 15,750 to 1575 descriptors (10%) preserved and slightly improved performance (macro-F1 from 0.4758 to 0.4977), indicating that discriminative information is concentrated in a compact subset of ordinal patterns. Two mechanisms may generate uninformative components within the GWOP representation. First, rarely visited ordinal patterns—particularly for D = 5, where 120 patterns are estimated from a single one-second window—yield per-segment probabilities based on few occurrences, so that their variance across segments is dominated by estimation noise rather than by class differences. Second, adjacent (q,τ) configurations produce strongly correlated descriptors, so the effective dimensionality of the representation is considerably lower than 15,750. Gain-based selection therefore does not merely produce a more compact representation, it also discards confounding descriptors that are dominated by estimation noise and which can actively mislead the classifier.

Class-wise ablation reveals two distinct behaviors. Rhonchi and Wheeze reach high F1 scores even with very small feature subsets. Their metrics are already substantial at 0.1% of the descriptors and saturate with more features. Both are continuous adventitious sounds with sustained, quasi-periodic oscillatory structure. A few ordinal configurations appear sufficient to describe this regularity. Normal segments behave similarly, although at a lower overall level. Stridor and Coarse Crackle follow the opposite trend. Their metrics remain low below 1% of the features and improve markedly as the subset grows. Stridor shows the steepest change, with F1 rising from about 0.20 at 0.1% to values above 0.45 once 1–10% of the descriptors are retained. Its precision increases in the same direction, indicating that additional descriptors provide discriminative rather than merely redundant information. This asymmetry suggests that the number of ordinal descriptors required is class-dependent rather than a global property of the representation. A similar behavior has been reported in sleep stage characterization, where stages with stereotyped dynamics are separable with compact ordinal descriptions, whereas transitional stages require richer representations [32].

The parameter composition of the top-10% subset reported in Table 4 is dominated by D=5 (74% of top features), with a non-uniform distribution of temporal delays. The proportion of selected descriptors increases monotonically from 15% at τ=1 to 25% at τ=5. This suggests that respiratory sound discrimination benefits from ordinal relationships sampled over wider temporal spans rather than from strictly consecutive samples. The analysis of the entropic index *q* offers particularly insightful findings. While q=0 was frequently selected for fine temporal scales, the broad presence of both positive (q=3,7,9) and negative (q=−2,−8) values demonstrates that discriminative information is distributed across different fluctuation regimes. Positive *q* values emphasize high-amplitude fluctuations (wheezes, rhonchi), whereas negative values enhance low amplitude fluctuations (fine crackles, normal breathing texture). This complementary sensitivity provides direct empirical support for the generalized entropy framework and confirms that different respiratory sound categories operate at distinct amplitude scales.

Comparison with MFCC features further substantiates the informational advantage of GWOP. Improvements in accuracy (from 42% to 50%) and Cohen’s kappa (from 0.2874 to 0.3923) indicate that ordinal dynamics capture complementary information beyond the spectral envelope. The largest gains occur in Stridor and Coarse Crackle, the smallest in Wheeze_Crackle. This ordering is consistent with what each representation encodes. MFCCs summarise the spectral envelope over analysis frames, which suits sounds whose identity resides in a stable spectral signature. Stridor and crackles are instead defined by their temporal morphology. Brief, sharply delimited events whose spectral average over a one-second window are easily dominated by the surrounding breathing sound. Ordinal descriptors operate on the local rank structure of the waveform and are therefore less affected by this averaging. The improvement in Stridor precision is particularly relevant in this respect (from 0.3636 to 0.7931), since it indicates that the ordinal representation supplies a positive signature for the class rather than merely relaxing a decision threshold. GWOPs reduce confusion between Coarse Crackle and Wheeze from 31% to 15%, and increase Stridor recognition from 20% to 38%. These are clinically significant improvements, as crackles and stridor are key indicators of interstitial lung diseases and upper airway obstruction, respectively.

The subject-wise ablation test also provided meaningful information about the nature of adventitious noises. Model quality degradation was uneven among classes. In four of them (Fine Crackle, Normal, Rhonchi and Whezze) there were only marginal changes. In Wheeze Crackle and Coarse Crackle, the model quality diminished, and in Stridor the model was completely unable to perform detections. This behavior was observed both in the GWOP and MFCC classifiers, which suggests the existence of class-specific subject-wise trends in the data that affect the behavior of both models. Another significant consideration that emerges from this ablation test is that the classes in which the performance dropped are the least prevalent (with the notable exception of Rhonchi), which may suggest that in larger datasets the fingerprinting effects may be mitigated.

In interpreting these results, the pediatric nature of the SPRSound dataset should be considered. Respiratory sound characteristics are known to vary across developmental stages due to differences in thoracic dimensions, respiratory rate, and chest wall thickness, which affect both normal and adventitious sounds. Since the proposed descriptors are selected based on their statistical association with abnormal sound events rather than age specific spectral profiles, they capture general acoustic patterns of adventitious sounds as they manifest in the SPRSound population (ages 1 month to 18 years). This provides internal variability within the pediatric range (and even among different age groups) but does not guarantee generalization to adult populations. External validation on adult respiratory sound datasets would therefore be required before the proposed representation can be claimed to generalize beyond pediatric recordings.

## 5. Conclusions

This work explored the applicability of Generalized Weighted Ordinal Pattern probability distribution as a feature representation for the characterization of adventitious respiratory sounds. Rather than summarizing the ordinal information into a single entropy value, the proposed approach directly features the complete probability distribution, preserving richer information about the nonlinear temporal dynamics of respiratory signals. The discriminative capability of this representation was evaluated using XGBoost as a classification model, while the XGBoost Gain criterion was employed as an analytical tool to investigate the contribution of individual descriptor components. Results demonstrated that the proposed GWOP representation provides a more informative characterization than conventional MFCC features under the same evaluation protocol. Furthermore, the Gain-based analysis showed that the discriminative information contained in the high-dimensional GWOP feature space is concentrated in a compact subset of descriptors. Beyond the classification results, the proposed framework provides a systematic way to analyze the temporal organization of respiratory signals through weighted ordinal dynamics, offering insights that are not directly available from conventional spectral representations.

Despite these findings, respiratory sound characterization remains a challenging problem due to the high intra-class variability and acoustic similarity among several categories. Future studies should evaluate the generalization capability of the GWOP representation on different databases and acquisition protocols. Related work implies that GWOP may achieve valuable acquisition-independent results [35]. Also, it is worth to investigate the combination of GWOP with complementary representations or temporal modeling approaches across the complete respiratory cycle to further explore the information contained in the sound dynamics. Finally, since the most informative descriptors arose at the highest embedding dimension (D=5), further investigation should focus on the performance of even higher embedding dimensions (D=6,7). However, this should be carefully balanced against the factorial growth of the ordinal pattern space and the increasing sparsity of the GWOP representation.

## Figures and Tables

**Table 1:** Class balancing process and final class distribution. Majority classes (N, W, FC) were balanced by random downsampling, whereas minority classes (CC, R, S, WC) were not augmented and retained their original sample sizes.

Class	Original	Operation	Final
N	5096	Random downsampling	2038
W	4307	Random downsampling	1722
FC	5080	Random downsampling	2032
CC	654	–	654
R	917	–	917
S	306	–	306
WC	1322	–	1322
Total	17,682	–	8991

**Table 2:** Five-fold cross-validation results for the GWOP feature representation.

Fold	Acc	F1-mac	F1-w	Prec (mac)	Rec (mac)	Spec (mac)	AUC
1	0.4663	0.4594	0.4648	0.5693	0.4320	0.9051	0.8194
2	0.4892	0.4848	0.4867	0.5646	0.4588	0.9094	0.8228
3	0.4701	0.4572	0.4677	0.5545	0.4312	0.9061	0.8278
4	0.4569	0.4527	0.4558	0.5302	0.4276	0.9036	0.8147
5	0.4652	0.4315	0.4567	0.4988	0.4203	0.9056	0.8200
Mean	0.4695	0.4571	0.4663	0.5435	0.4340	0.9060	0.8210
Std	0.0120	0.0190	0.0125	0.0292	0.0146	0.0021	0.0048

**Table 3:** Ablation analysis of the GWOP representation using top-*N* (by feature count, ranked by XGBoost Gain) feature selection. Results are obtained on the held-out test set. For each Gain threshold, *N* indicates the number of retained GWOP features for each embedding dimension (*D*), while D% represents the percentage of retained features relative to the complete feature set of that dimension.

Total Features	*D*	*N*	F1	Spec.	Prec.	Sens.	AUC
15,750 (100%)	3	630/630	0.4758	0.9086	0.5665	0.4507	0.8336
	4	2520/2520					
	5	12,600/12,600					
7875 (50%)	3	423/630	0.4749	0.9082	0.5593	0.4509	0.8352
	4	1657/2520					
	5	5795/12,600					
3150 (20%)	3	170/630	0.4904	0.9105	0.5735	0.4648	0.8356
	4	666/2520					
	5	2314/12,600					
1575 (10%)	3	78/630	0.4977	0.9112	0.5695	0.4732	0.8366
	4	327/2520					
	5	1170/12,600					
788 (5%)	3	43/630	0.4910	0.9101	0.5522	0.4673	0.8304
	4	170/2520					
	5	575/12,600					
315 (2%)	3	18/630	0.4953	0.9090	0.5629	0.4699	0.8249
	4	79/2520					
	5	218/12,600					
158 (1%)	3	9/630	0.4820	0.9086	0.5288	0.4637	0.8180
	4	42/2520					
	5	107/12,600					
79 (0.5%)	3	7/630	0.4513	0.9032	0.4831	0.4393	0.8042
	4	19/2520					
	5	53/12,600					
16 (0.1%)	3	2/630	0.3413	0.8920	0.3436	0.3521	0.7365
	4	4/2520					
	5	10/12,600					
8 (0.05%)	3	1/630	0.3152	0.8888	0.3164	0.3299	0.7045
	4	2/2520					
	5	5/12,600					
4 (0.025%)	3	0/630	0.2728	0.8820	0.2717	0.2949	0.6730
	4	1/2520					
	5	3/12,600					

**Table 4:** Distribution of the top 1575 most informative GWOP features (top 10% by feature count) identified by XGBoost Gain analysis. For each embedding dimension (*D*), the table reports the contribution of each dimension, the distribution of temporal delays (τ), and the dominant values of the entropic index (*q*).

Dimension	τ	Freq.	Dominant *q* Values
D=5 (74%)	1	15%	q=0 (11%), q=1 (10%), q=8 (8%), q={2,3,4,7,9} (7% each), remaining *q* values (~3% each).
	2	18%	q={0,2} (9% each), q=1 (7%), q={3,6,7,8,9,10} (6% each), q={−9,−7,5} (5% each), remaining *q* values (~3% each).
	3	18%	q={0,4} (7% each), q={−7,1,2,5,9,10} (6% each), q={−5,−4,6} (5% each), q={−9,−8,−3,3,7,8} (4% each), remaining *q* values (~2% each).
	4	23%	q=0 (9%), q=2 (8%), q={1,3,10} (7% each), q={5,6,9} (6% each), remaining *q* values (~3% each).
	5	25%	q=0 (13%), q=1 (10%), q=10 (7%), q={2,3,4,9} (6% each), remaining *q* values (~3% each).
D=4 (21%)	1	22%	q=1 (14%), q=0 (11%), q={3,10} (8% each), q={2,9} (7% each), remaining *q* values (~4% each).
	2	20%	q=1 (18%), q=0 (17%), q={−2,2,8,9} (6% each), q={−8,−5,3,6} (5% each), remaining *q* values (~3% each).
	3	21%	q=0 (16%), q={1,5} (11% each), q=8 (10%), q=10 (7%), remaining *q* values (~4% each).
	4	16%	q=0 (17%), q=2 (15%), q=8 (13%), q=4 (10%), remaining *q* values (~4% each).
	5	21%	q=1 (14%), q=2 (13%), q=0 (10%), q={7,8,10} (9% each), remaining *q* values (~4% each).
D=3 (5%)	1	22%	q=0 (24%), q={−10,−9,1,2,10} (12% each), q={−5,−4,−1} (6% each).
	2	24%	q=1 (21%), q=0 (16%), q={−6,2,3,6} (11% each), q={−9,−7,−5,7} (5% each).
	3	19%	q=1 (27%), q={0,2,7,9} (13% each), q={−8,4,5} (7% each).
	4	21%	q={1,4} (25% each), q=2 (19%), q=5 (12%), q={−6,0,3} (6% each).
	5	14%	q={2,3,6} (18% each), q={−7,0,4,5,10} (9% each).

**Table 5:** Comparison between MFCC and GWOP feature representations using the optimized XGBoost classifier as a common evaluation model. In all cases the later performed better.

Class/Metric	MFCC			GWOP		
	Prec.	Rec.	F1	Prec.	Rec.	F1
CC	0.4878	0.1527	0.2326	0.6667	0.3053	0.4188
FC	0.3733	0.3440	0.3581	0.4344	0.4963	0.4633
N	0.4110	0.4755	0.4409	0.4874	0.5196	0.5030
R	0.4901	0.4044	0.4431	0.6497	0.5574	0.6000
S	0.3636	0.1967	0.2553	0.7931	0.3770	0.5111
W	0.4234	0.4725	0.4466	0.5623	0.4841	0.5202
WC	0.4474	0.5795	0.5050	0.4579	0.6174	0.5258
Test Accuracy	0.4202			0.5053		
Balanced Accuracy	0.3750			0.4796		
Cohen’s Kappa	0.2874			0.3923		
AUC Macro	0.7860			0.8383		

**Table 6:** Subject-wise partition: number of subjects and segments per age group in the training and test sets.

Group	Age (Years)	Subjects			Segments	
		Train	Test	Total	Train	Test
G1	0–2	60	15	75	1148	599
G2	2–4	117	29	146	1825	639
G3	4–6	184	46	230	1746	464
G4	6–8	125	31	156	1275	133
G5	8–10	66	16	82	334	183
G6	10–12	45	11	56	431	39
G7	≥12	31	8	39	141	34
Total		627	156	783	6900	2091

**Table 7:** Subject-wise partition: number of segments per class in the training and test sets.

Class	Train	Test	Test Fraction
CC	501	153	23%
FC	1555	477	23%
N	1603	435	21%
R	638	279	30%
S	214	92	30%
W	1315	407	24%
WC	1074	248	19%
Total	6900	2091	23%

## Data Availability

The dataset used in this study, SPRSound, is publicly available at https://github.com/SJTU-YONGFU-RESEARCH-GRP/SPRSound (accessed on 1 March 2026). No new primary data were collected; the derived feature sets generated during this study are available from the corresponding author upon reasonable request.

## Supplementary Materials

The following supporting information can be downloaded at: https://www.mdpi.com/article/10.3390/e28101092/s1, Figure S1: Confusion matrices in absolute sample counts for (a) the baseline and (b) the optimized XGBoost classifier using the GWOP representation. Normalized versions are shown in Figure 4 of the main text. Figure S2: Confusion matrices in absolute sample counts for (a) the complete GWOP feature representation (100%, 15,750 features) and (b) the top-10% Gain-selected subset (1575 features), ranked by individual feature Gain. Normalized versions are shown in Figure 7 of the main text. Figure S3: Confusion matrices in absolute sample counts for (a) the conventional MFCC representation and (b) the GWOP representation with Gain-based top-10% feature selection, both evaluated with the optimized XGBoost classifier. Normalized versions are shown in Figure 8 of the main text.
