# Supplementary material for: Generalized Weighted Ordinal Pattern Descriptors for the Characterization of Adventitious Respiratory Sounds
Source: Entropy (Basel). 2026 Oct 7;28(10):1092. doi: 10.3390/e28101092 (PMC13703545; doi:10.3390/e28101092)
Supplement: Supplementary file 1 [file entropy-28-01092-s001.zip › entropy-4559454-supplementary.pdf]

Supplementary Materials: Generalized Weighted Ordinal Pattern Descriptors for the Characterization of Adventitious Respiratory Sounds

Jorge I. Lopez Perez, Cristina D. Duarte and Claudio Delrieux

This supplementary section reports the confusion matrices of the main text in absolute sample counts on the held-out test, complementing the normalized versions shown in Figures 4, 7 and 8 of the main text.

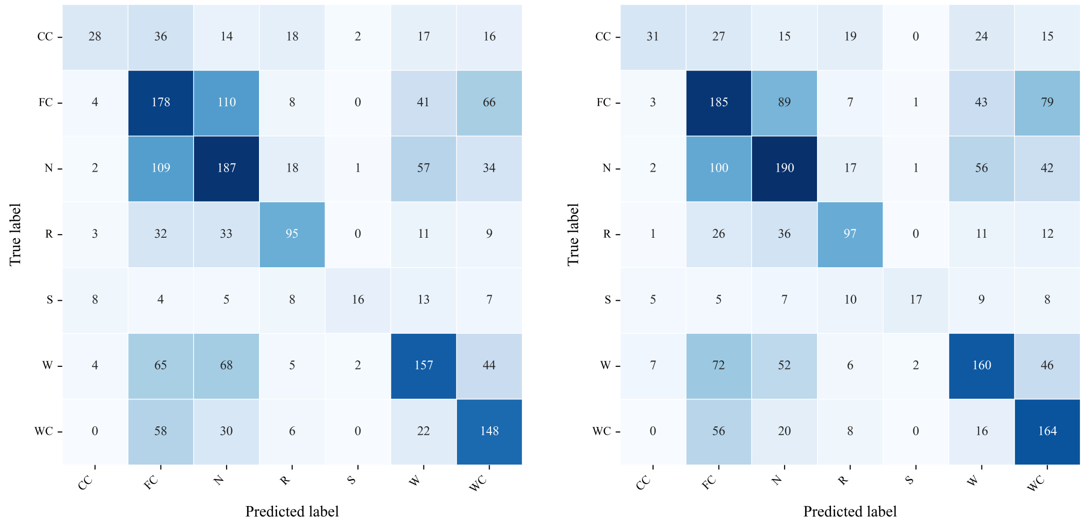

(a) (b)

Figure S1. Confusion matrices in absolute sample counts for (**a**) the baseline and (**b**) the optimized XGBoost classifier using the GWOP representation. Normalized versions are shown in Figure 4 of the main text.

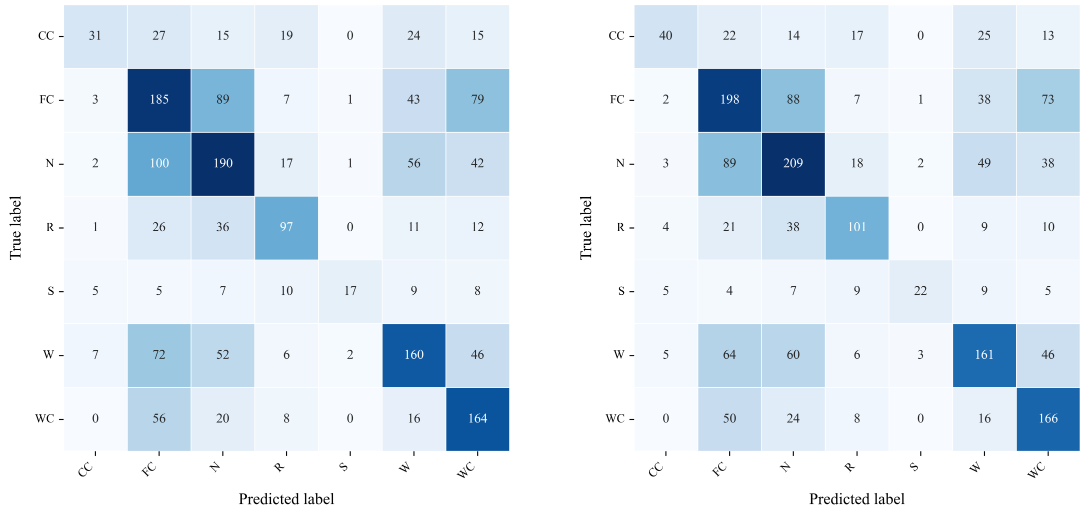

(a) (b)

Figure S2. Confusion matrices in absolute sample counts for (**a**) the complete GWOP feature representation (100%, 15,750 features) and (**b**) the top-10% Gain-selected subset (1575 features), ranked by individual feature Gain. Normalized versions are shown in Figure 7 of the main text.

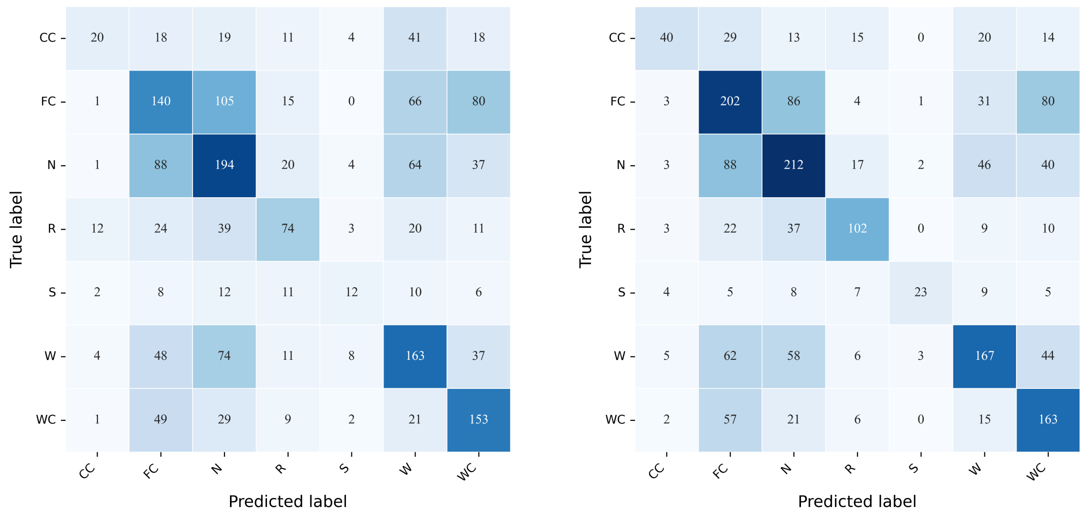

(a) **(b)**

Figure S3. Confusion matrices in absolute sample counts for (**a**) the conventional MFCC representation and (**b**) the GWOP representation with Gain-based top-10% feature selection, both evaluated with the optimized XGBoost classifier. Normalized versions are shown in Figure 8 of the main text.
